# Tasseled Crop Rows Detection Based on Micro-Region of Interest and Logarithmic Transformation

**DOI:** 10.3389/fpls.2022.916474

**Published:** 2022-06-27

**Authors:** Zhenling Yang, Yang Yang, Chaorong Li, Yang Zhou, Xiaoshuang Zhang, Yang Yu, Dan Liu

**Affiliations:** ^1^School of Engineering, Anhui Agricultural University, Hefei, China; ^2^Institute of Artificial Intelligence, Hefei Comprehensive Nation Science Center, Hefei, China; ^3^Faculty of Artificial Intelligence and Big Data, Yibin University, Yibin, China; ^4^JD AI Research, Beijing, China

**Keywords:** agricultural machinery navigation, crop rows detection, micro-region of interest, energy-efficient, logarithmic transformation

## Abstract

Machine vision-based navigation in the maize field is significant for intelligent agriculture. Therefore, precision detection of the tasseled crop rows for navigation of agricultural machinery with an accurate and fast method remains an open question. In this article, we propose a new crop rows detection method at the tasseling stage of maize fields for agrarian machinery navigation. The whole work is achieved mainly through image augment and feature point extraction by micro-region of interest (micro-ROI). In the proposed method, we first augment the distinction between the tassels and background by the logarithmic transformation in RGB color space, and then the image is transformed to hue-saturation-value (HSV) space to extract the tassels. Second, the ROI is approximately selected and updated using the bounding box until the multiple-region of interest (multi-ROI) is determined. We further propose a feature points extraction method based on micro-ROI and the feature points are used to calculate the crop rows detection lines. Finally, the bisector of the acute angle formed by the two detection lines is used as the field navigation line. The experimental results show that the algorithm proposed has good robustness and can accurately detect crop rows. Compared with other existing methods, our method's accuracy and real-time performance have improved by about 5 and 62.3%, respectively, which can meet the accuracy and real-time requirements of agricultural vehicles' navigation in maize fields.

## 1. Introduction

In recent years, advances in intelligent agriculture have effectively reduced human costs and decreased the human harm caused by chemical factors such as pesticides. Real-time navigation of machines walking in the field is crucial for agriculture. Among them, the most popular approaches for field navigation are still path planning based on Global Position System (GPS) (Jin and Tang, 2011; Hameed, 2014; Li et al., 2019; Wang et al., 2021) and machine vision-based navigation (Ball et al., 2016; Radcliffe et al., 2018; Mavridou et al., 2019; Rovira-Mas et al., 2021; Vrochidou et al., 2022). The development of path planning algorithms has solved the path tracking problem of agricultural machinery on a global scale, but the phenomenon of seedling injury from wheels is still inevitable. Since crops are usually sown in rows, machine vision-based field navigation is a promising way to provide navigation paths for agricultural machinery. Among them, the critical technology of computer vision, feature extraction (Manavalan, 2020; Xue et al., 2020, 2021a; Shrivastava and Pradhan, 2021; Vishnoi et al., 2022), is widely used in crop rows detection due to its advantages, such as low reliance on data resources. Many researchers have devoted significant efforts to developing efficient field navigation algorithms, and they can be classified into the following types.

### 1.1. Methods Based on Hough Transform

Hough (1962) proposed a way to transform points from a right-angle coordinate system into hough space. It has a good performance in processing information with straight-line features, but the real-time performance and accuracy of crop rows detection are unsatisfactory with mid-late stage plants. Thus, various improvements have been proposed. Ji and Qi (2011) detected crop rows by randomly selecting feature points for the Hough transform and then using many-to-one mapping to parameter space. Gall et al. (2011) established Hough Forest to improve the speed of Hough straight line detection. Winterhalter et al. (2018) proposed a reliable plant splitting pipeline and detected crop rows by Hough transform, but this approach is still limited to the crop rows at the early stage.

### 1.2. Methods Based on Horizontal Strips

It is very difficult to extract crop information from non-parallel crop rows in the image. This problem is effectively solved by dividing the image into multiple horizontal strips and processing them in successive steps. Ma et al. (2021) determined the number of crop rows by dividing horizontal strips in the upper part of the image. Ospina and Noguchi (2019) derived detection lines of crop rows by dividing horizontal strips. Crop contours in each strip are calculated, and their geometric centers are extracted as feature points for fitting. Zhou et al. (2021) determined the multi-ROI by dividing the horizontal strips. The initial ROI is calculated and continuously slides upward for the update. Finally, the midpoints of each ROI are fitted to make a navigation line. This method does not fully extract crop information when dealing with discrete characteristics of plants.

### 1.3. The Deep Learning Method

During the past decades, deep neural networks (DNNs) have made great success in field detection. Bah et al. (2020) combined Convolutional Neural Networks (CNN) and the Hough transform to detect crop rows in the field. Adhikari et al. (2020) used a deep network to learn semantic images, which makes the input images directly output detection lines as tractor control signals. Lac et al. (2022) first used a deep neural network to detect the plant stem and then used an aggregation algorithm to refine the localization of the crop further. Ponnambalam et al. (2020) divided the image into a vehicle driving area and a crop area using semantic segmentation based on CNN, and feature points are extracted. They further fitted the feature points derived from the multi-ROI to plan the crop rows detection lines. Although DNNs have good performance in accuracy, they really require large computing resources, and this limits their practical applications (Zhang et al., 2018a; Roy et al., 2019; Pan et al., 2021).

### 1.4. Integrated Approaches

Yu et al. (2021) proposed a treble classification and two-dimensional clustering-based crop rows detection in paddy fields for the problem of numerous weeds and floating weeds in the paddy fields. This method used a triple Otsu's (Otsu, 2007) method approach for segmentation and fitted the detection lines after selecting the misleading points by a two-dimensional adaptive clustering method. This method needs to be improved in terms of real-time performance. Jiang et al. (2015) integrated the crop rows with close geometric features in the robot walking area by multi-ROI for optimization and extraction of the crop rows centroids by clustering method. The detection lines were extracted by the linear regression method. Tenhunen et al. (2019) segmented the green objects after segmentation and obtained the direction and distance information between crop rows using a two-dimensional Fourier transform, then performed a clustering operation and finally obtained the location of the crop row. This algorithm is still deficient in coping with strong illumination conditions. Rabab et al. (2021) investigated adaptive crop row detection in variable field environments without the need to determine the number of crop rows by clustering. The method has good adaptability. Zhang et al. (2018b) defined clusters of feature points and fitted crop rows detection lines through a clustering algorithm and optimal path selection.

### 1.5. Our Contributions

In this study, we propose a new crop rows detection method for real-time navigation in maize fields during the tasseling stage. The article makes the following main contributions.

(1) To solve the difficulty of segmentation caused by the concentrated distribution of each color component in the image, we propose an image enhancement method based on logarithmic transformation, which well increases the contrast between the tassels and the background.

(2) We propose a method to determine ROI (Montalvo et al., 2012) by two steps of approximate selection and update, which overcomes the problem that ROI cannot achieve adaptivity in extracting information from skewed crop rows images.

(3) To verify the performance of the proposed method, various experiments are conducted to analyze the effect of parameters and make a comparison with the existing related studies. The experimental results demonstrate the advantages of this study in terms of accuracy and real-time performance.

**Figure 1:**
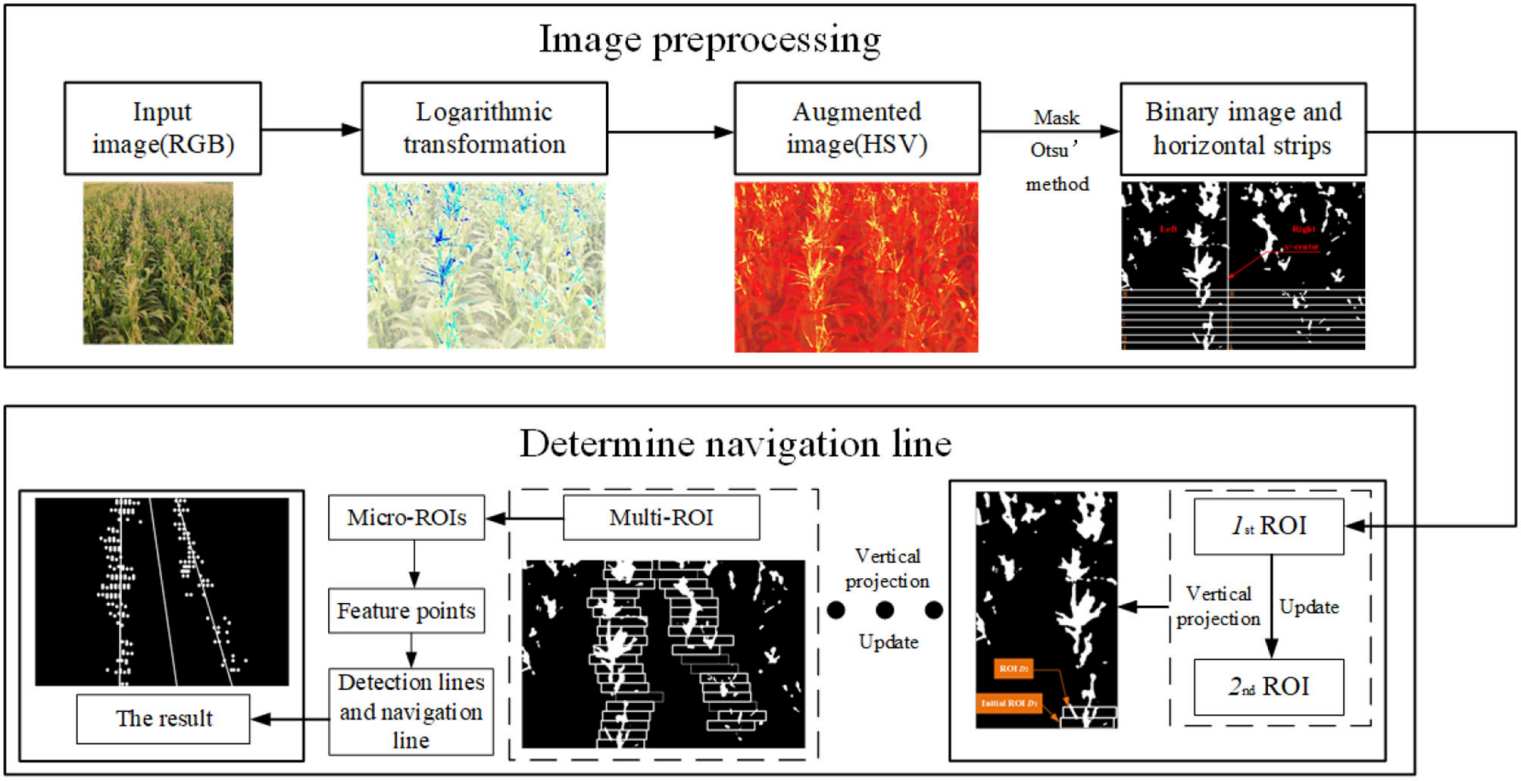
Flowchart of the whole extract navigation line algorithm.

Specifically, this study demonstrates the possibilities and prospects of feature extraction algorithms for extracting navigation lines in the tasseled maize field. We have overcome the problems of complex tassels segmentation and non-adaptive ROI. The whole process of this algorithm is shown in Figure 1. Our proposed model consists of two main parts: image preprocessing and determination of navigation lines. In image preprocessing: First, the logarithmic transformation is applied to augment this capture images. Second, these images are transformed into HSV space and segmented the tassels from the background by grayscaling and Otsu's method. Finally, the binarised image is morphologically processed to decrease impulse noise. In the part of the determination navigation line: First, we divide the image into multiple horizontal strips, and then the initial ROI is determined using the bounding box. Second, continuously slide up the bounding box and update it until the whole image is completed to get the multi-ROI. Then, dividing the micro-ROIs to extract the feature points. Finally, the detection lines of the crop rows are made by using the least square method to fit these points. We further compute the bisector of the acute angle formed by the two detection lines and use it as the navigation line.

## 2. Methods

While the deep learning-based methods achieve good performance in image processing and crop rows detection, they usually require a lot of computing resources, such as graphics processing units and GPU clusters. Based on image enhancement, selection of ROI, and delineation of horizontal strips, we propose an accurate and fast method for crop rows detection in the maize field during the tasseling stage. This section will introduce two main modules of our proposed method: image pre-processing and the determination of the navigation line.

### 2.1. Image Pre-processing

#### 2.1.1. Pixel Value Modification

The distribution between crop rows in the upper part of the image is very concentrated, making it challenging to distinguish crop rows when extracting crop information. Additionally, the upper part of the image is not very meaningful for navigation; we only need the lower part of the image as the navigation area. Thus, the lower 3/4 of the image is taken as the processing object, and then the image is partially cropped to remove the redundant information. Finally, the size of the pixel value of the image is modified to 600 × 600 pixels.

#### 2.1.2. Logarithmic Transformation

The grayscale processing (Liu et al., 2010, 2020; Laursen et al., 2014) is mainly performed using the excess green (ExG) (Comba et al., 2015; Tang et al., 2016; Chen et al., 2020) feature operator when segmenting the crops with the background at the early stage. However, ExG was not effective in processing maize at the tasseling location. The logarithmic function shows a nonlinear feature is uniformly increasing the independent variable. Additionally, the magnitude of change gradually decreases. Based on this, we adopt the logarithmic transformation for the pixel values of each pixel point in the image under RGB color space, which can augment the distinction between the tassels and background and realize the segmentation of the picture. When establishing the logarithmic function, the following factors are considered: (1) Transformation should avoid negative results after the logarithmic operation; (2) The effect of differentiation between the tassels and the background is augmented after the logarithmic operation. The logarithmic function established is Equation (1).


(1) $$\begin{array}{r} Out=C\times log\left(1.0+p\right), \end{array}$$

where *Out* is the result of the pixel value operation of the pixel point; *p* is the pixel value of the pixel point in the image to be processed; and *C* is a constant.

#### 2.1.3. Building Masks and Morphological Processing

The images calculated in the way of 2.1.2 are transformed from RGB to HSV color space. We will determine the suitable threshold to build the mask through subsequent experiments. The mask images are converted to grayscale images, and Otsu's method (Cellini et al., 2017) is performed to extract the tassels. At the end of this part, it is necessary to select the appropriate kernels for the morphological processing of the image.

### 2.2. Determination Navigation Line

In this section, we describe how to determine a navigation line. It mainly contains two parts: selecting ROIs and planning navigation lines.

#### 2.2.1. Select ROIs

The algorithm addresses the problem of navigation line extraction for field vehicles. We believe that the crop rows in the traveling area (the two crop rows in the center of the image) are valid for navigation, while the crop rows at the image boundary can be disregarded. Due to the perspective principle, the crop rows are not parallel in the image, bringing more significant difficulties to feature point extraction. Therefore, we extracted feature points by selecting ROIs.

**Figure 2:**
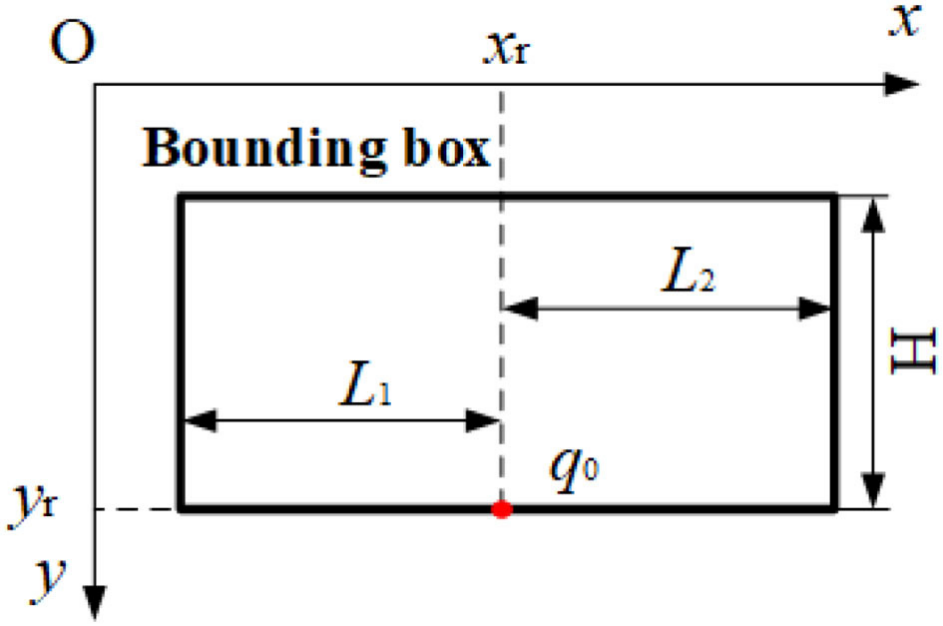
The structure of the bounding box.

To determine the ROI of an image, we specify a bounding box, which is described as follows: The coordinates of point *q*_0_ *(Xr, Yr)* are used as the origin, *L1+L2* (*L1*=45pix, *L2*=55pix) as the width and *H* (20pix) as the height to determine the bounding box shown in Figure 2. Since *L1, L2*, and *H* are all constants and only the coordinates of the center point *q*_0_ are variable. Thus, the location of the bounding box is expressed in Equation (2).


(2) $$\begin{array}{r} B=\left(X_{r},Y_{r}\right). \end{array}$$

##### 2.2.1.1. Divide the Image Band

**Figure 3:**
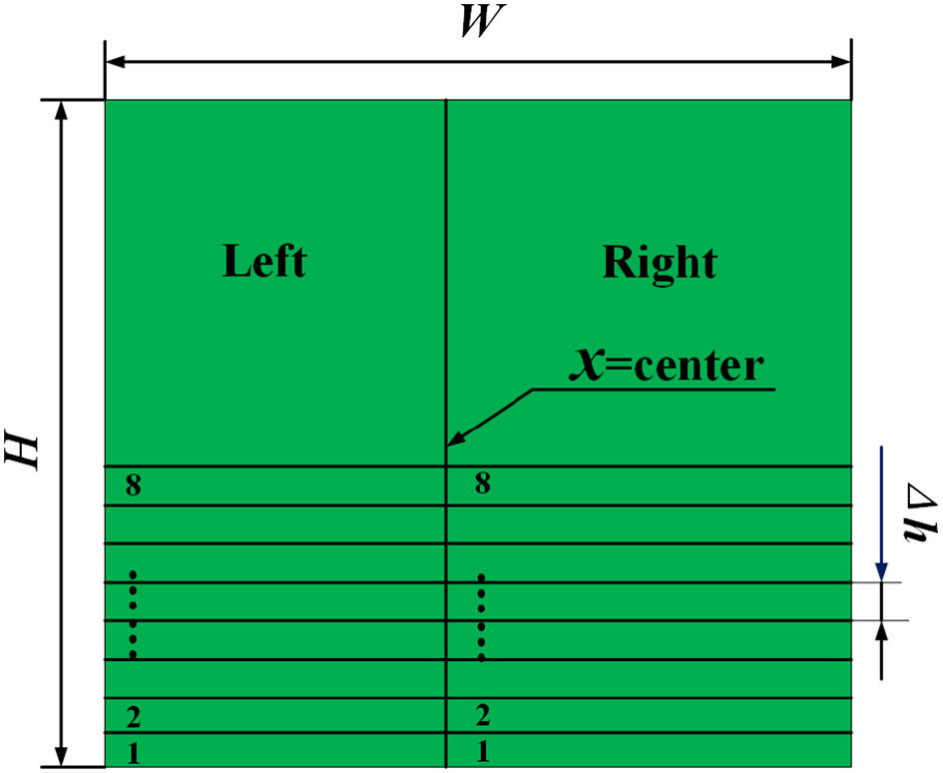
Image division processing. The green part is the binarized image. *W* and *H* is the image pixel value of width and high.

We divide the binarized images according to the following way: The image is divided into left and right areas using the line *x*=center (260pix) as the dividing line. The areas are labeled as *Left* and *Right*. The algorithm uses the same approach for *Left* and *Right*, thus, we only describe the process of *Left* in the following step. On *Left*, eight horizontal strips are divided in step length of Δ*h* (20pix) from bottom to top, each strip was numbered *Ks* (s=1,2,3...,8). The resulting model is shown in Figure 3.

The unstable environment such as light and weeds makes the work hard of getting crop rows information, we determine the ROI by approximate selection and update. The primary choice is completed first. The bounding box is applied to frame the approximate position of the initial ROI. Then this ROI is updated according to its local pixel distribution to obtain a more accurate result of the initial ROI.

We start by approximately selecting the initial ROI with a bounding box, marking it as *B*_1_, and the process is as follows: First, we set the threshold *Y=15*. The cumulative value *M(j)* of the number of white pixels in each column of the strip is calculated using Equation (3) sequentially in the order of the labels until the maximum value of *M(j)* ≥ *Y* in the *Ks* strip, and this strip is name *K*_*j*_. The values of columns whose coordinates are higher than *Y* are counted, and it is stored in list *Q*. In *Q*, the closest value to the *center* is the horizontal coordinate of the origin of the initial bounding box, and the lower boundary of the strip is the vertical coordinate of the origin of the initial bounding box. The coordinates of the origin of the bounding box are given by Equation (4), then *B*_1_ is framed.


(3) $$\begin{array}{r} M\left(j\right)=\frac{\sum_{i=1}^{\Delta h}p_{\left(i,j\right)}=255}{255},j\in \left\{1,2,...,w\right\}, \end{array}$$

where *j* is the column coordinate of the pixel point of the image strip; *i* is the row coordinate of the pixel point of the image strip; *p(i,j)* is the pixel value of the coordinate; *w* is the horizontal pixel size of the strip.


(4) $$\begin{array}{r} \left(j_{s},W-\left(K_{s}-1\right)\times △h\right). \end{array}$$

##### 2.2.1.2. The Method of Updating Bounding Box

To achieve a more accurate location of the ROI, we propose an updated method, and the steps are as follows.

(1) First, the number of white pixels in each column of the bounding box is counted using Equation (5). Mark the horizontal coordinate of the lower boundary of the bounding box as *x*_*p*_ (*x*_*p*_=1,2,3..., *L1+L2*). The distribution of white pixels in the bounding box for the part of the horizontal coordinate less than *x*_*p*_ and more than *x*_*p*_ are expressed respectively as Equation (6) and Equation (7).


(5) $$\begin{array}{r} Z\left(v\right)=\frac{\sum_{u=1}^{\Delta h}p_{\left(u,v\right)}=255}{255},v\in \left\{1,2,...,L1+L2\right\}, \end{array}$$

where *u* is the row coordinate of the pixel point within the ROI; *v* is the column coordinate of the pixel point within the ROI; *p*_(*u, v*)_ is the pixel value of this point.


(6) $$\begin{array}{r} I_{l}=\overset{x_{p}\;}{\underset{v=1}{\sum }}Z\left(v\right)\times \left(x_{p}-v\;\right), \end{array}$$

(7) $$\begin{array}{r} I_{r}=\overset{L1+L2\;}{\underset{v=x_{p}+1}{\sum }}Z\left(v\right)\times \left(v-x_{p}\;\right), \end{array}$$

where *Z(v)* is calculated by Equation (5) *v* is the column coordinate of the pixel point of the image strip; *L1+L2* is the width pixel value of the bounding box.

(2) To find a point in the bounding box that makes the distribution of pixels balanced, we build an evaluation function *f(x*_*p*_*)*, which is shown as Equation (8). For the second-order partial derivative (Equation (9) of *f(x*_*p*_*)*. Since Equation (9) >0, there is a minimum value of *f(x*_*p*_*)*. The horizontal coordinates of the bounding box are updated to the horizontal coordinates of the lowest point of *f(x*_*p*_*)* which is determined by Equation (10), and the vertical coordinates are unchanged.


(8) $$\begin{array}{r} f\left(x_{p}\right)=|I_{l}-I_{r}|, \end{array}$$

(9) $$\begin{array}{r} \frac{\partial^{2}f\left(x_{p}\right)}{\partial x_{p}^{2}}=2, \end{array}$$

(10) $$\begin{array}{r} j_{k}=argminf\left(x_{p}\right). \end{array}$$

##### 2.2.1.3. Determining the Final Result of the Initial ROI

By means of 2.2.1.2 [Due to the white pixels of *B*_1_ are already calculated by Equation (3) and do not need to be repeated by Equation (5)], the bounding of *B*_1_ is updated, and the coordinate origin of the result is *(j*_*k*1_*, W-(K*_*s*_*-1)* × Δ*h)* [*j*_*k*1_ is the result of the calculation of Equation (10)]. This bounding box determines the final outcome of the initial ROI, marked as *D*_1_.

##### 2.2.1.4. Sliding of Bounding Box

After obtaining the initial ROI, the ROI named *B*_2_ is determined by sliding the bounding box of the initial ROI in the negative direction along the y-axis in step length Δ*h*. The coordinate of the bounding box origin of *B*_2_ is *(j*_*k*1_*, W-K*_*s*_ × Δ*h)*. *T*_1_ is the number of white pixel points inside *B*_2_, calculated by Equation (11).


(11) $$\begin{array}{r} T_{1}=\overset{L1+L2}{\underset{n=1}{\sum }}\overset{\Delta h}{\underset{b=1}{\sum }}p\left(b,n\right)=255, \end{array}$$

where *b* is the row coordinate of the pixel point; *n* is the column coordinate of the pixel point. *p(b,n)* is the value of the pixel point in *B*_2_.

The algorithm updates the bounding box of the optimized *B*_2_ to obtain the updated result *D*_2_ by establishing a threshold *T*_0_. We take *T*_0_ as 20 and offset *d*=20 in this article. The updated result *D*_2_ for *B*_2_ is derived and framed. The updating method is as follows:

(1) *T*_1_<*T*_0_: *B*_2_ is judged to be a sparse crop area. Considering that the distribution of the crop rows in the image is skewed, we slide the bounding box of *B*_2_ in the image coordinate system along the x-axis toward the line *x*=center by sliding step of offset *d*, and the result of D2 is determined.

(2) *T*_1_≥*T*_0_: *B*_2_ is determined as a feature region. The bounding box of *B*_2_ is updated by the method of 2.2.1.3 to give the coordinates of the origin as *(j*_*k*2_*, W-K*_*s*_ × Δ*h)*, and the result frame *D*_2_ [*j*_*k*2_ is the result of the calculation of Equation (10)].

##### 2.2.1.5. Determination of Multi-ROI

There is a tendency for the crop rows to converge in the image. Based on this feature, the steps in 2.2.1.4 are repeated. The process is as follows. First, The bounding box of the current ROI(*D*_*e*_) (subscript e is the number of the ROI, *e*=1,2,3...) is slid up to determine the subsequent ROI (*B*_*e*+1_) in the strip. Then *B*_*e*+1_ is updated and optimized to obtain. *D*_*e*+1_ until the multi-ROI is reached.

#### 2.2.2. Planning Navigation Line by Getting Micro-ROIs

The part marked as *Right* are processed using the methods in 2.2.1.1, 2.2.1.3, 2.2.1.4, and 2.2.1.5 in sequence. Therefore, all the ROI of the whole picture are determined.

This algorithm extracts feature points by building micro-ROIs. The method is as follows: Each ROI (*D*_1_, *D*_2_,...) is divided into 10 x 2 grids, each with a 10(pix) × 10(pix) micro-ROI. We set the threshold for the number of white pixels in the micro-ROI *H*_0_=20 and calculate the number of white pixels *H*_*n*_ in the individual micro-ROI. When *H*_*n*_ >*H*_0_, the geometric midpoint of the micro-ROI is a feature point.

Finally, these feature points are fitted using least squares to get the crop rows detection lines. The angle bisector at the intersection of the two identification lines is used as the navigation line.

## 3. Experimental Result and Discussion

In this section, we first introduce image acquisition. Then the results of each step are described. Finally, the performance of this article and existing algorithms is shown.

### 3.1. Image Acquisition and Processing Equipment

The subject of this study is the images of maize crops in the tasseling stage. The image acquisition device is a CMOS (Complementary Metal-Oxide-Semiconductor) machine vision camera, which is installed at a height of 2.9 m from the ground with a tilt angle of 20° the ground and calibrated using the camera imaging principle. The camera resolution is 1,920 × 1,080 pixels. Video is collected in Gu'an County, Hebei Province, China. The crops are planted at a row spacing of 60 cm, and the plant height was 2.7 m. The programming software was python 3.7 IDE, PyCharm professional 2020 compiler. The image processing hardware used Advantech MIC-7700 IPC, processor Intel Corei5, main frequency 2.5GHz, graphics card for NVIDIA GTX 1650, video memory 8G. The video was saved in AVI format. The video was collected on 8 July 2020 (illumination:101700 lx) and 11 July 2020 (illumination:130800 lx).

### 3.2. The Performance of the Proposed Model

**Figure 4:**
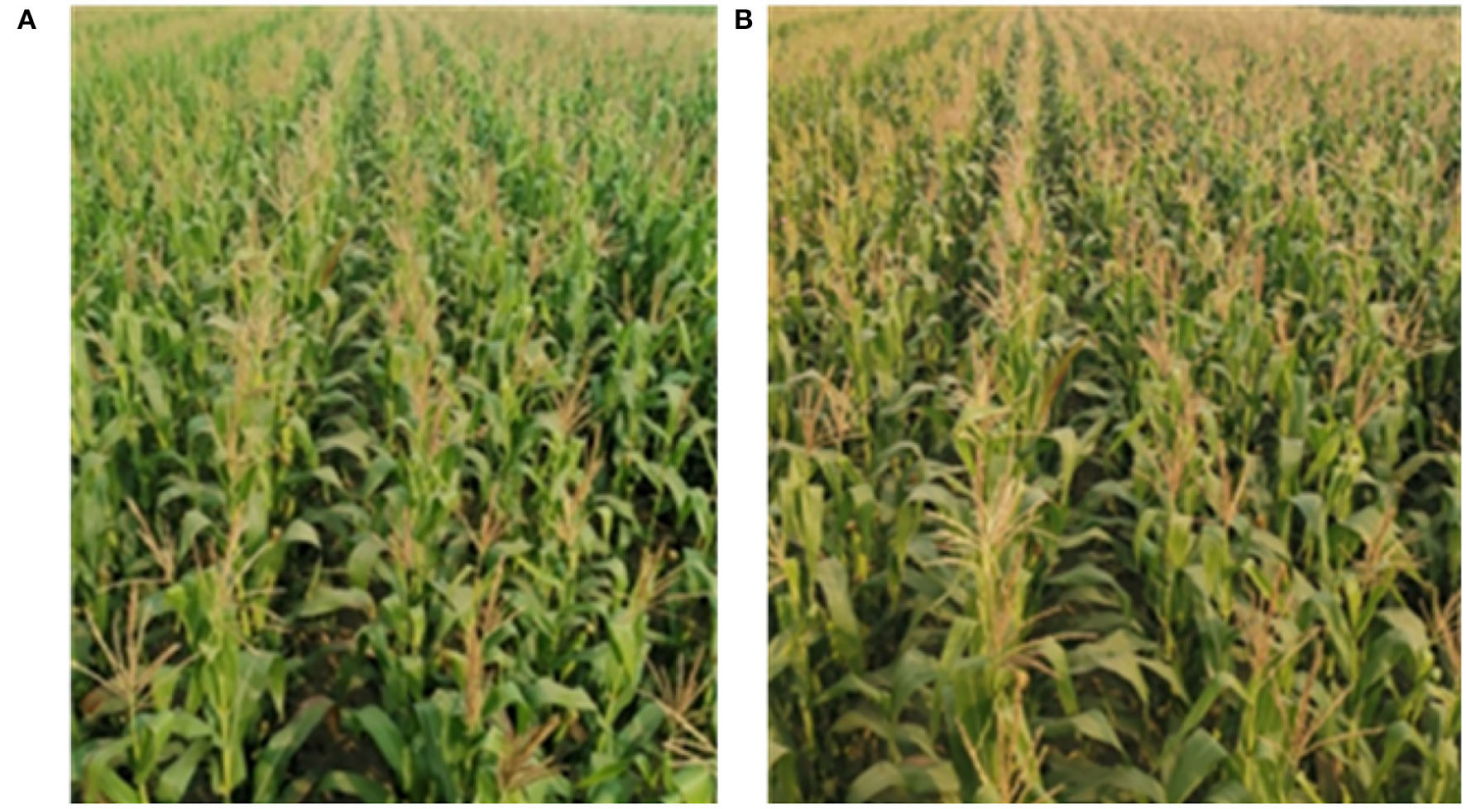
Image captured by CMOS. **(A)** Type-1 field (illumination: 130800 lx). **(B)** Type-2 field (illumination: 101700 lx).

In this part, we will use these images as examples to describe the result of every step. Example images are shown in Figure 4. In Figure 4, the illumination of the Type-1 field and Type-2 field are 130800 (lx) and 101700 (lx) respectively. First, the pixels of the two images are modified by way of 2.1.1. Then the results are processed in the following steps.

#### 3.2.1. The Result of Logarithmic Transformation

**Figure 5:**
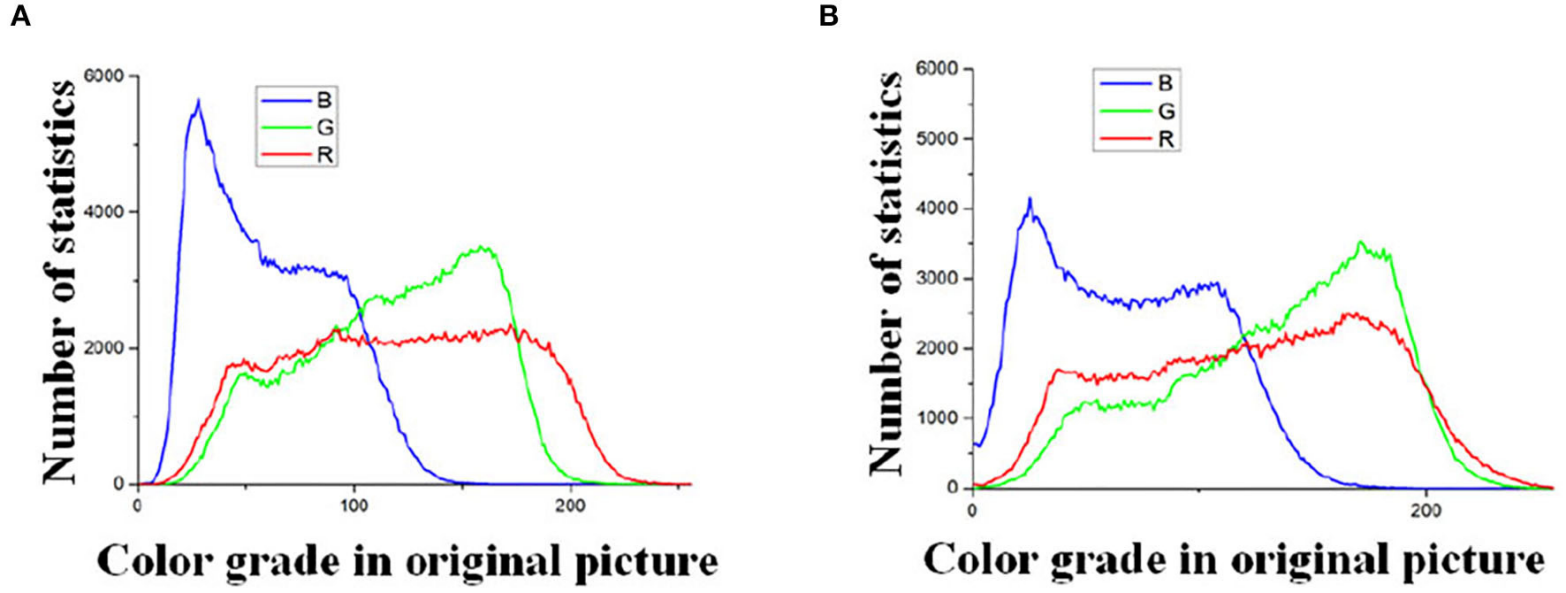
Statistics of color components in RGB space. **(A)** Color grade in original picture Type-1 field (illumination: 130800 lx). **(B)** Color grade in original picture Type-2 field (illumination: 101700 lx).

**Figure 6:**
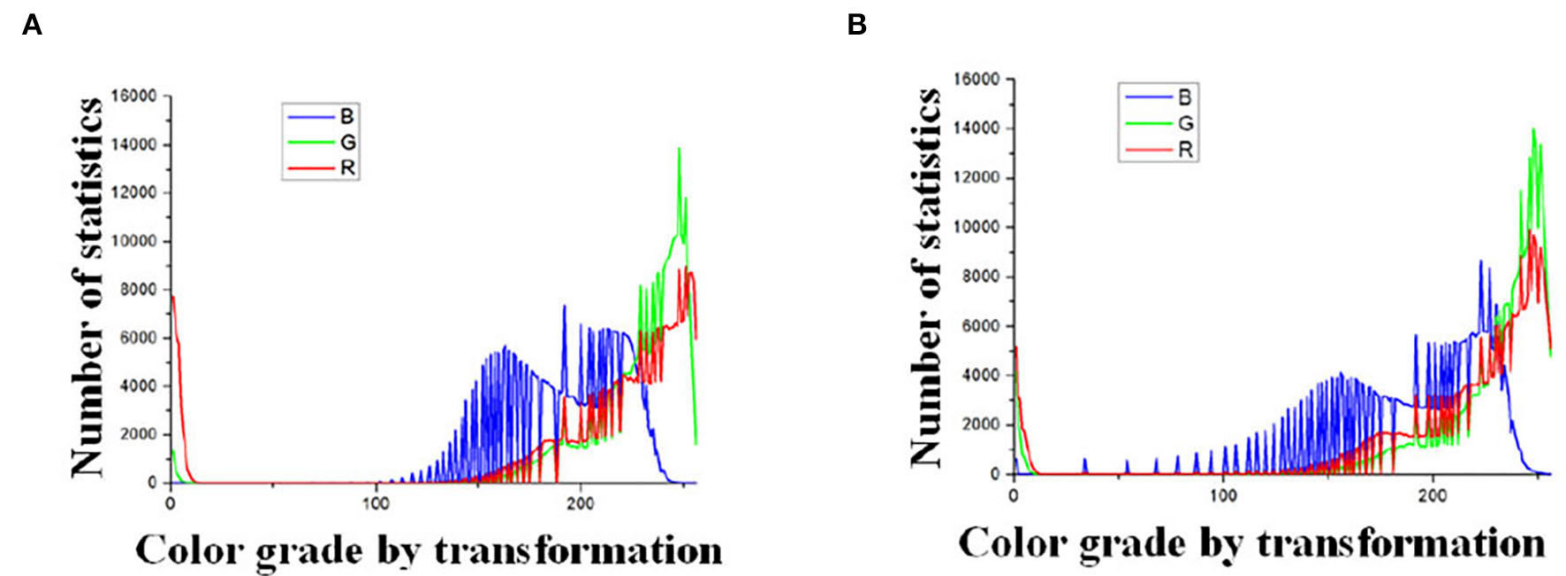
Statistics of color components after logarithmic transformation. **(A)** Color grade by transformation Type-1 field (illumination: 130800 lx). **(B)** Color grade by transformation Type-2 field (illumination: 101700 lx).

The processing results of Figure 4 are plotted with the frequency statistics of each color component (R, G, B) as shown in Figure 5. It can be found that the distribution of each color component is concentrated, indicating that the differentiation between the tassels and the backgrounds, such as leaves and soil, is not obvious under natural illumination, which makes it challenging for subsequent segmentation work. To determine the value of *C* in Equation (1), the experiment methods are as follows: We transformed the images by Equation (1), and the results of *C* taking values in the range 1 to 100 were observed. We found that the frequency distribution has clear discrimination when *C* is taken as 48. The statistical results are shown in Figure 6. Comparing the statistical results in Figures 5, 6, the application of logarithmic transformation significantly enhances the distinction between the tassels and the background, which provides the necessary conditions for the subsequent image segmentation, so we determine the value of *C* is 48.

#### 3.2.2. Result of Masks and Morphological Operations

**Figure 7:**
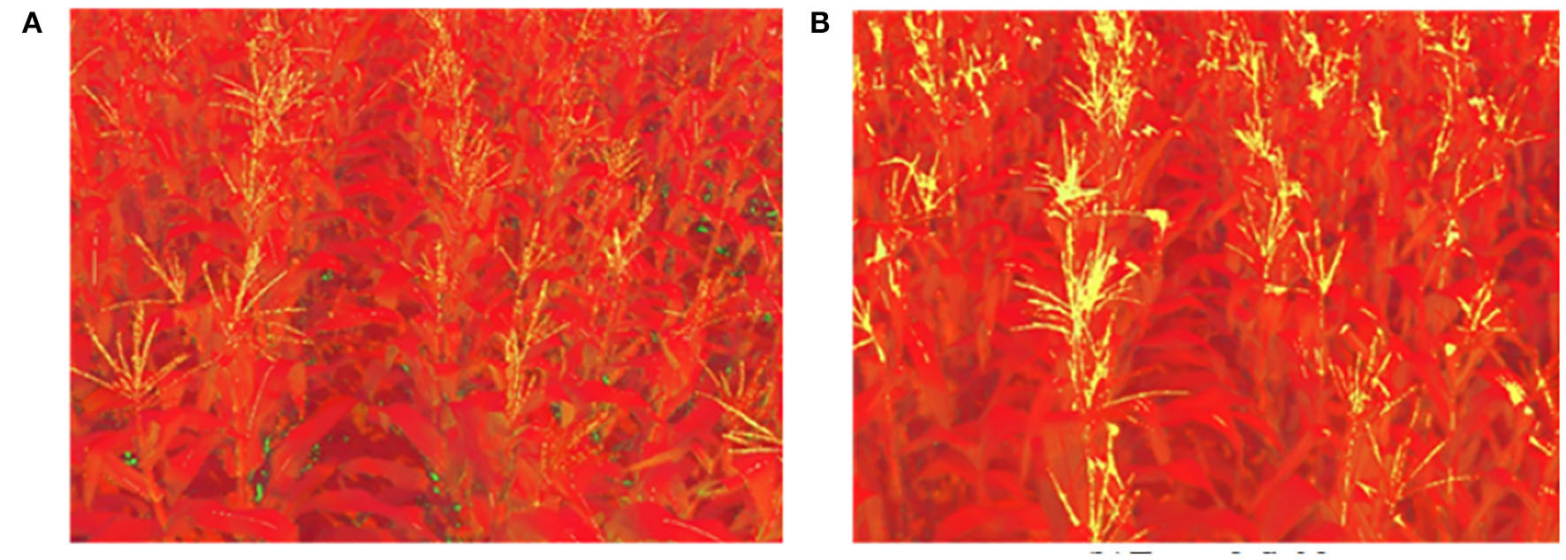
Images in HSV color space. **(A)** Type-1 field (illumination: 130800 lx). **(B)** Type-2 field (illumination: 101700 lx).

**Figure 8:**
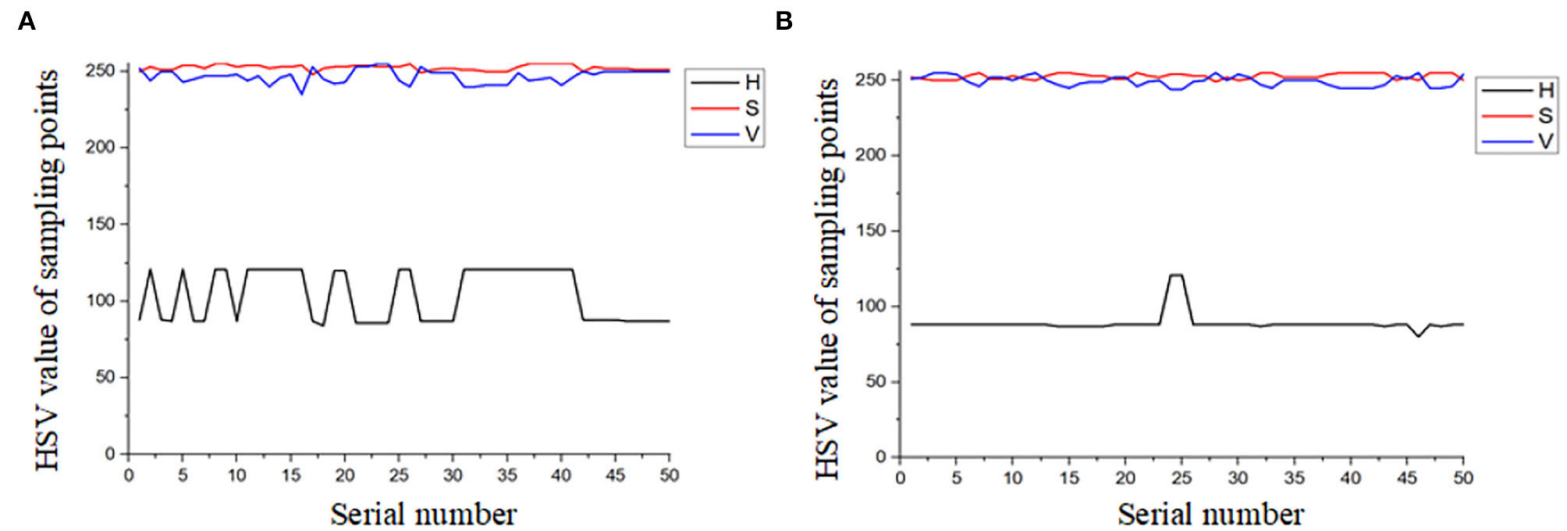
Distribution interval of H, S, and V at sampling points. **(A)** Type-1 field (illumination: 130800 lx). **(B)** Type-2 field (illumination: 101700 lx).

**Figure 9:**
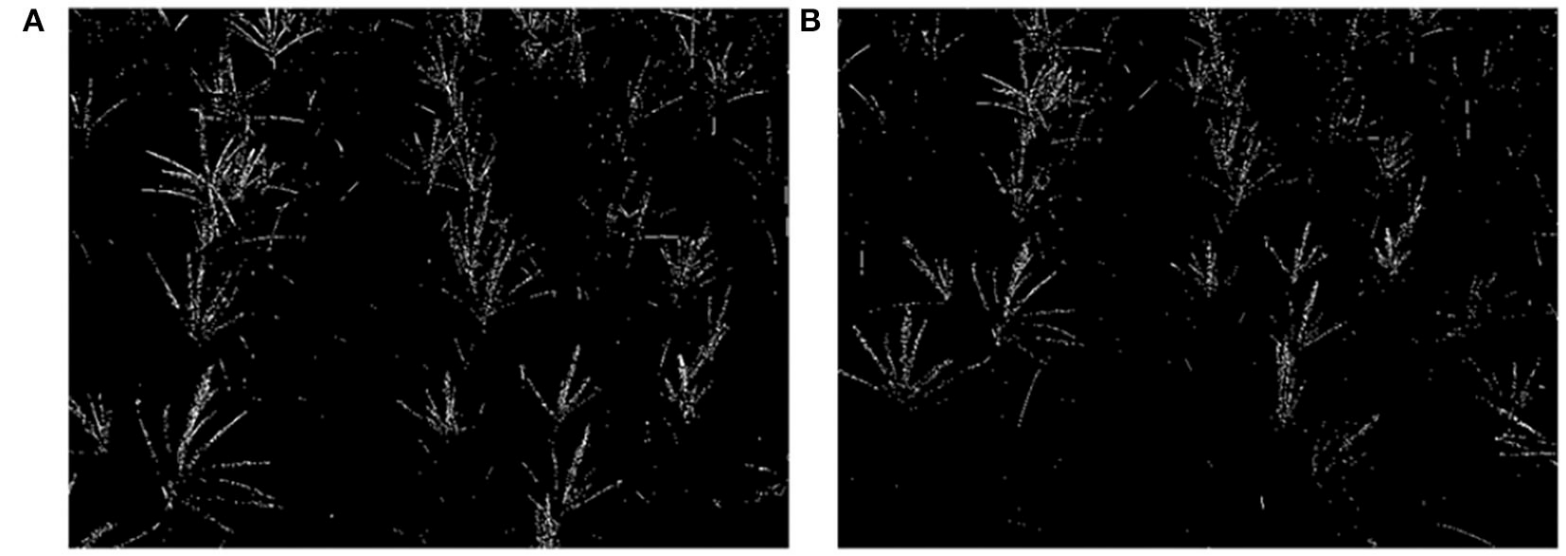
Binary images. **(A)** Type-1 field (illumination: 130800 lx). **(B)** Type-2 field (illumination: 101700 lx).

Images are transformed to HSV color space, and the result is shown in Figure 7. Fifty frames are randomly selected, and 50 sampling points are chosen on each of the tassels to count the distribution of H, S, and V. The statistical results are shown in Figure 8. Based on the statistical results, we set the threshold of *H* as [80,121], *S* as [250, 255], and *V* as [240, 255]. By these thresholds, the masks are built, mask images are transformed into grayscale images, and Otsu's method is performed to extract the tassels. The result is shown in Figure 9.

**Figure 10:**
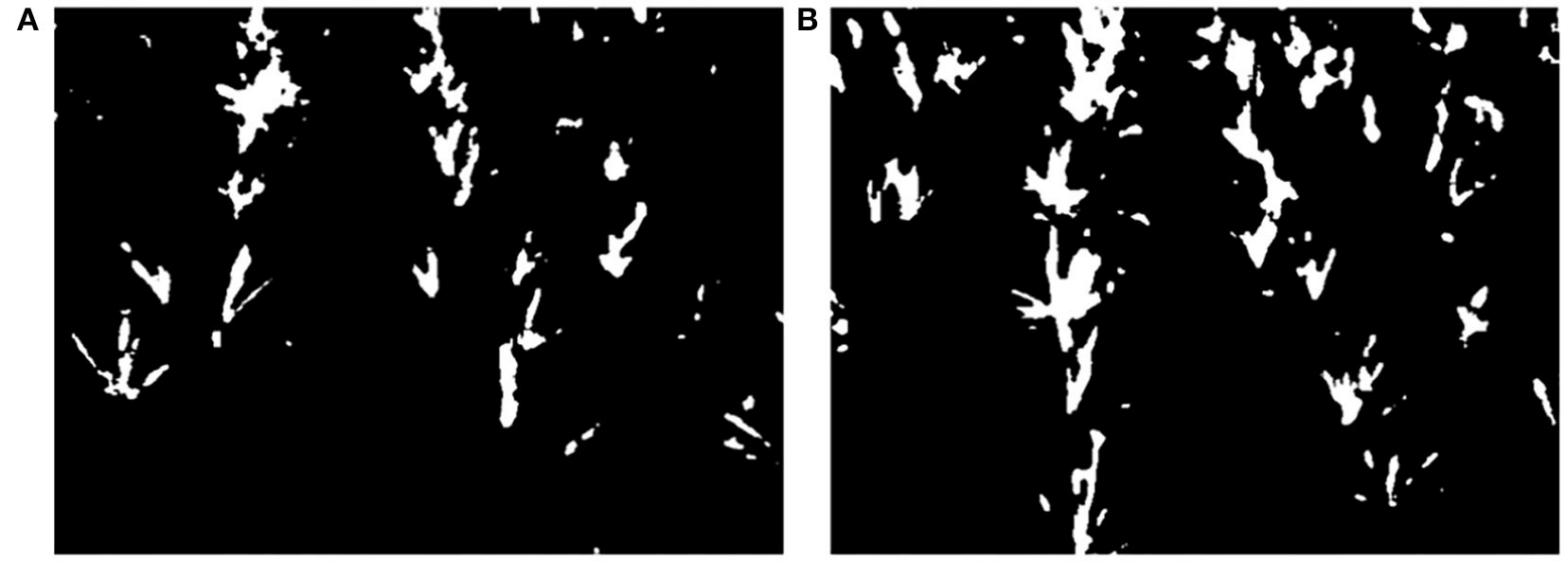
Results of morphological processing. **(A)** Type-1 field (illumination: 130800 lx). **(B)** Type-2 field (illumination: 101700 lx).

There is impulse noise in the binary images, and the feature pixel values of maize tassels are too small, making it challenging to extract feature points. It is necessary to perform morphological operations. Thus, we use a convolutional kernel of size 3 × 3 to inflate the image once morphologically. Then use a median filter with a convolutional kernel of size 9 to the noise for the inflated image. The morphological processing results are shown in Figure 10.

#### 3.2.3. The Result of Dividing Image, Initial ROI, and Second ROI

**Figure 11:**
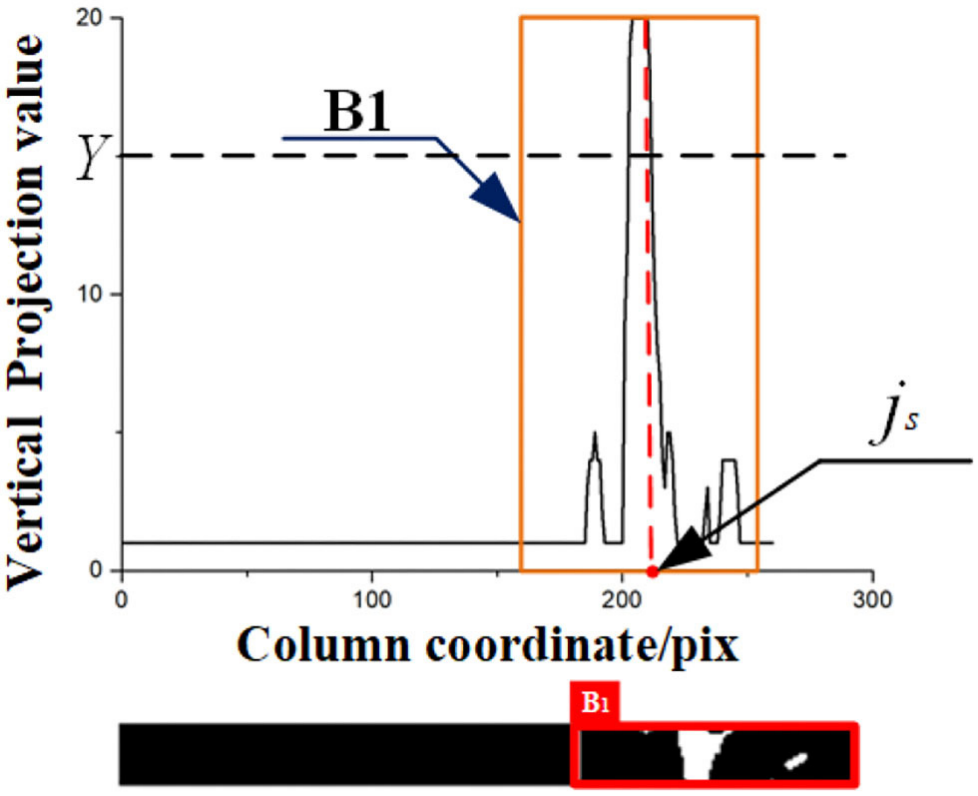
The projected image of the *K*_*j*_ strip (*K*_*j*_ is calculated in 2.2.1.1).

**Figure 12:**
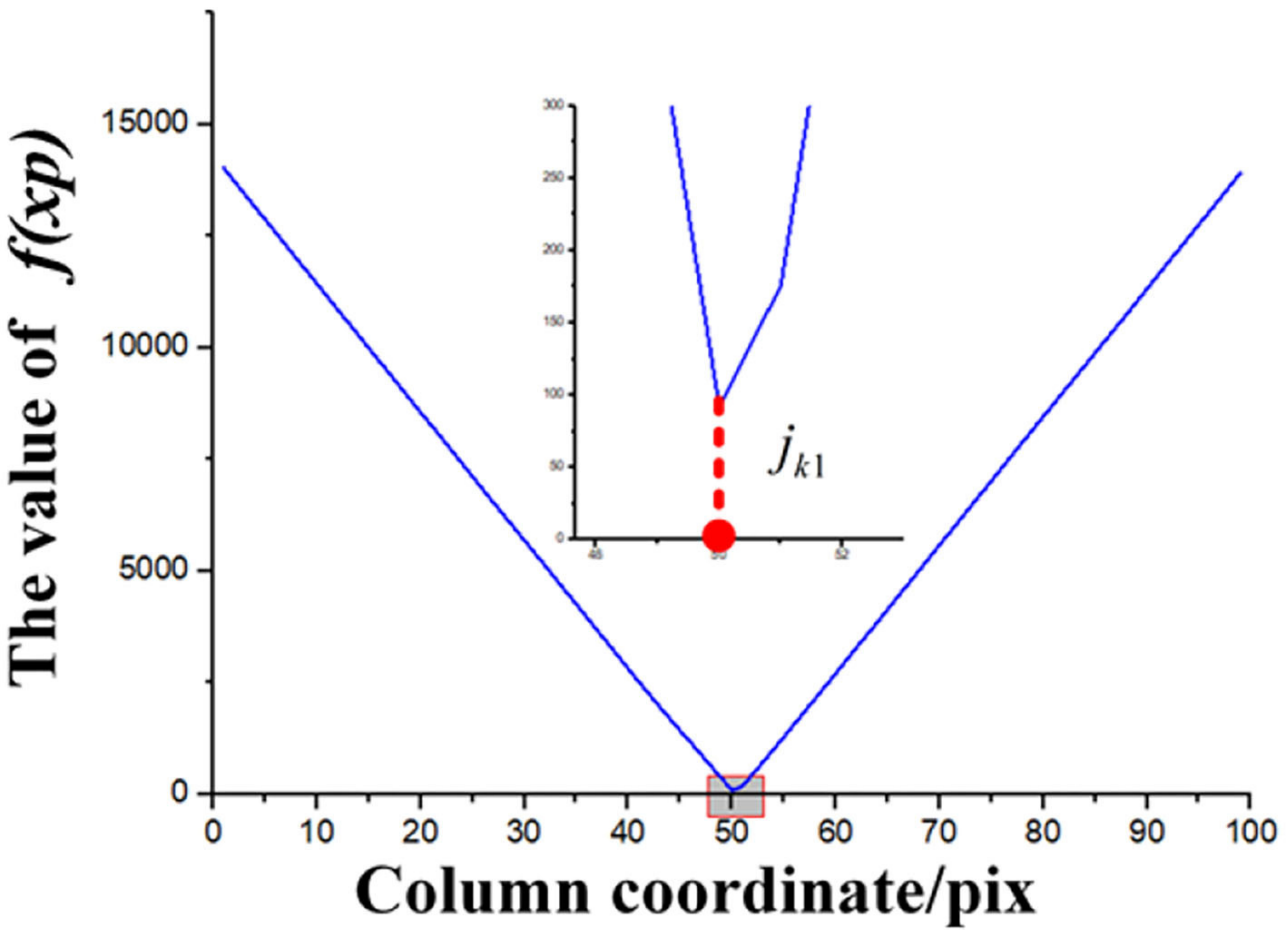
Distribution of the judging function.

**Figure 13:**
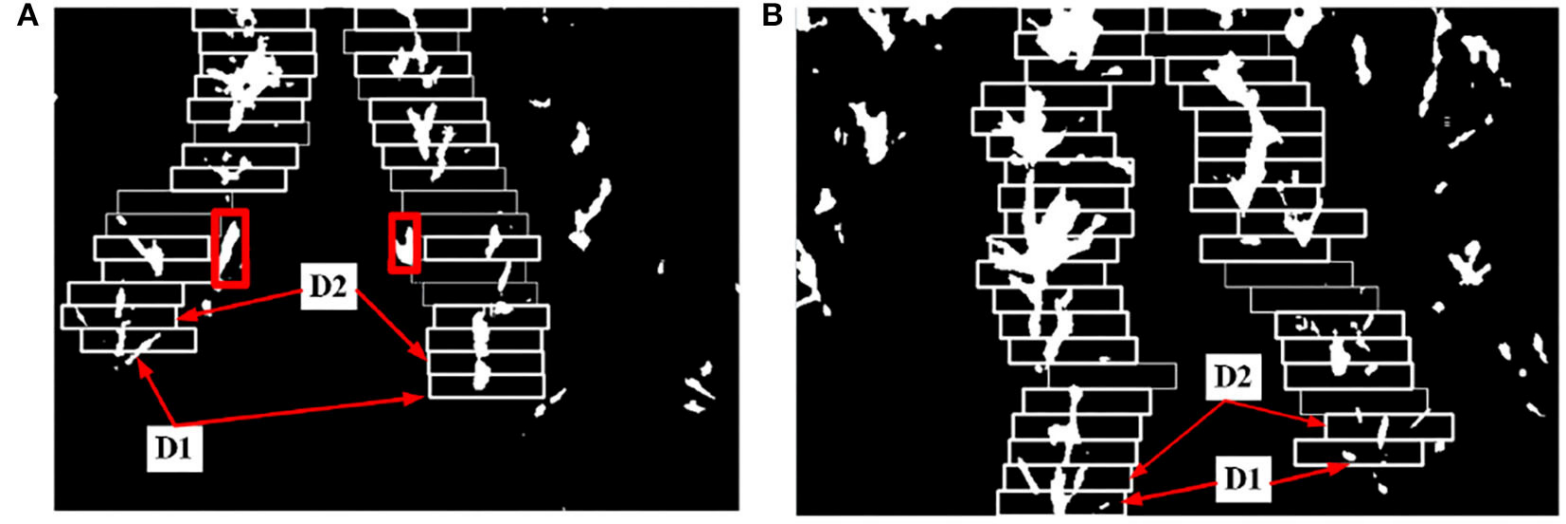
The result of multi-ROI extraction. **(A)** Type-1 field (illumination: 130800 lx). **(B)** Type-2 field (illumination: 101700 lx).

By the method of 2.2.1.1 to 2.2.1.3, the result of every step is as follows: Take Figure 10B as an example. We find the *k*_*s*_=1 horizontal strip. Its statistical graph of the number of white pixel dots in the column is shown in Figure 11. With the assistance of this graph, the rough initial ROI *B1* is determined, and the result is shown in Figure 11. By intercepting the curve inside *B*_1_ in Figure 11, we plot the distribution function of *f(x*_*p*_) as shown in Figure 12, and find the horizontal coordinate of the lowest point is *j*_*k*1_. The coordinates of the origin point of the bounding box are *(j*_*k*1_*, W-(K*_*s*_*-1)* × Δ*h)*. We use the bounding box to frame the final result of the initial ROI *D*_1_. Next, the second ROI can be determined by the method proposed in 2.2.1.4. The results are shown in Figure 13.

#### 3.2.4. The Result of Multi-ROI

The final multi-ROI can be determined by the method proposed in 2.2.1.5, the result is shown in Figure 13. The tassel feature pixels that deviate from the path appear during the upward sliding of the bounding box, as shown in the red box in Figure 13A. According to the original images, the pixels in this region correspond to tassels deviated from the crop rows, so this region is judged as deviated and will not be processed. Our proposed method allows the ROI always to follow the crop rows trend and guarantees the reliability of the subsequent feature point extraction work as much as possible.

#### 3.2.5. The Result of Feature and Navigation Line

**Figure 14:**
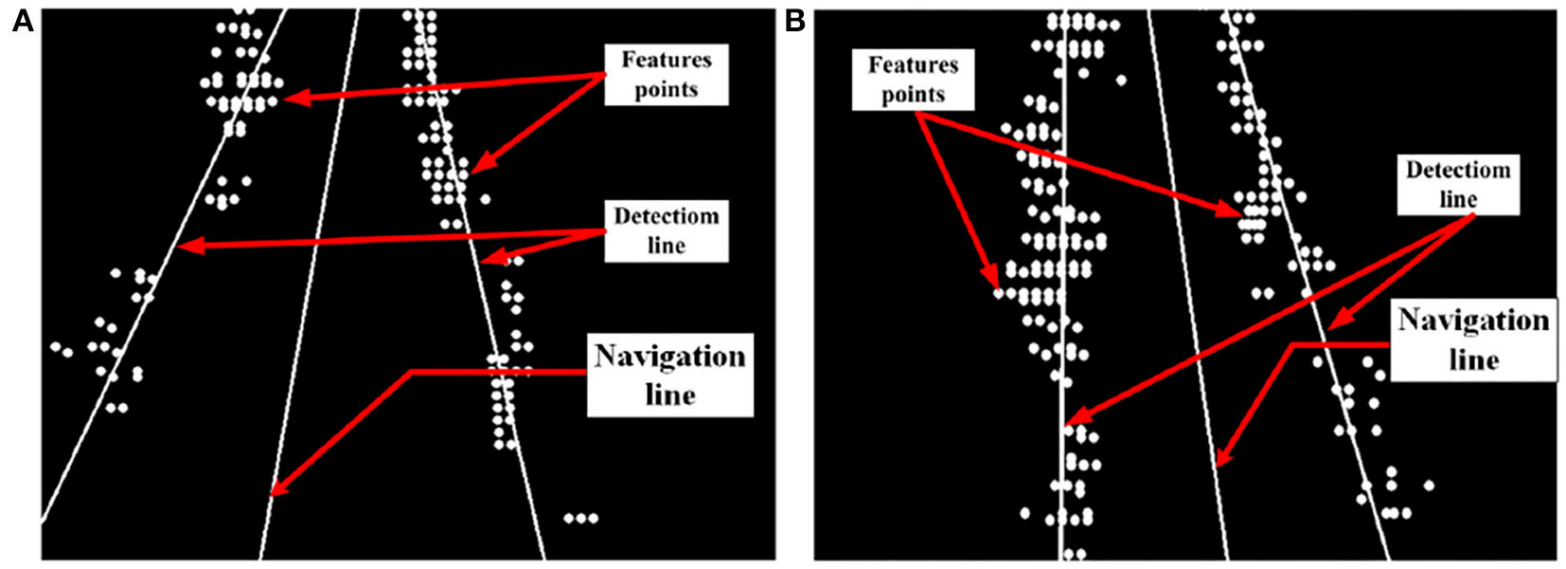
Fitting results for feature points. **(A)** Type-1 field (illumination: 130800 lx). **(B)** Type-2 field (illumination: 101700 lx).

By the method proposed in 2.2.2, feature points are extracted. We further plan the crop rows detection lines and the field navigation line. The result is shown in Figure 14.

### 3.3. Results and Discussion

**Figure 15:**
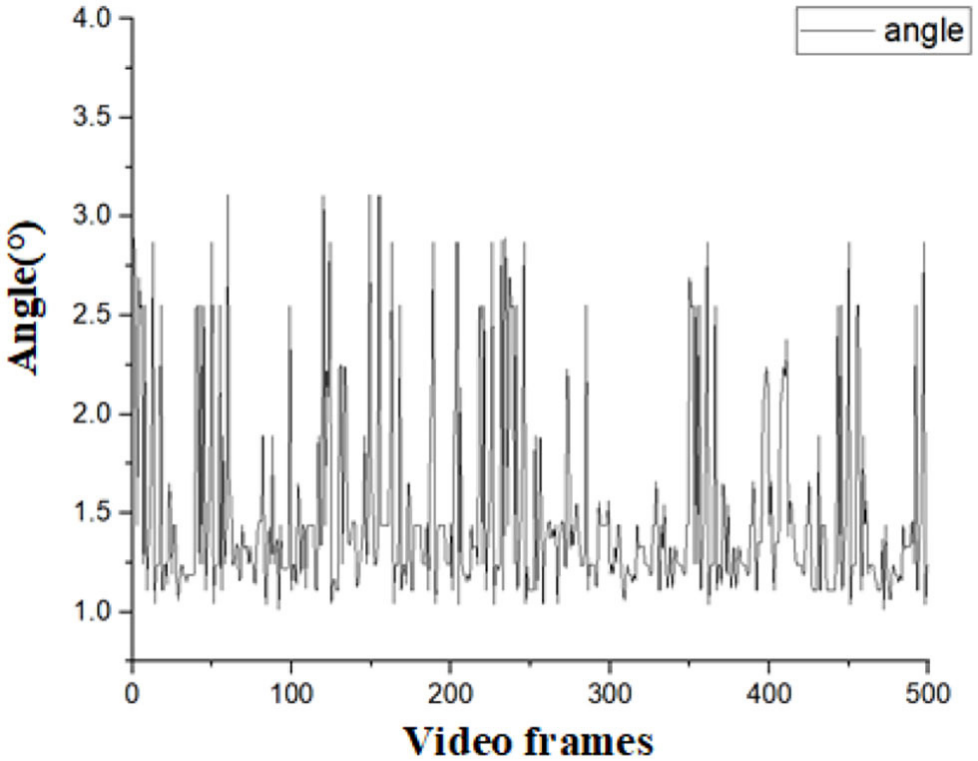
Calculated angles in the illumination of 101700(lx).

**Figure 16:**
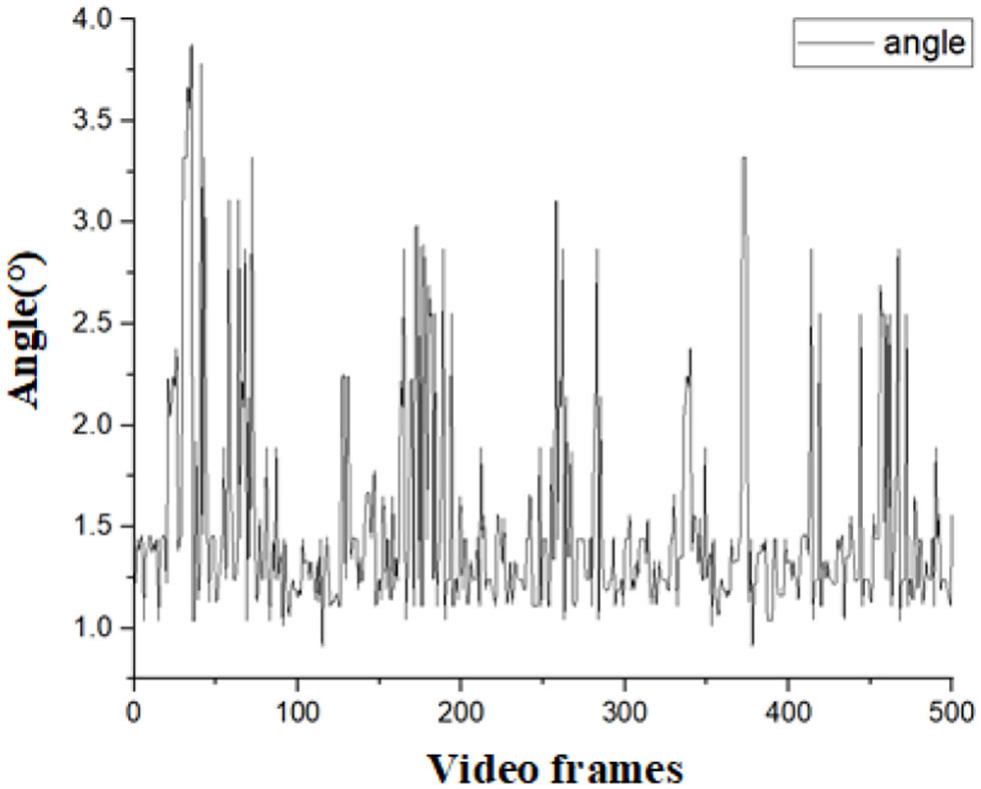
Calculated angles in the illumination of 130800(lx).

To verify the accuracy of the detection results. We designed an experiment as follows: Since the navigation line is calculated by crop rows detection lines, only the declination angle θ between the navigation line and the manually drawn navigation lines is needed to evaluate the detection accuracy of the algorithm. If θ is less than 5°, the detection result for navigation can be considered accurate. As the speed of agricultural machinery in the field is very slow, no more than 0.5m/s, we considered that the algorithm can meet the basic real-time requirements when the frames per second (FPS) is greater than 4. A total of 1,000 frames of video taken in the field were randomly selected for the experiment. Among this video, 500 frames are in 101700(lx) illumination, and others are in 130800(lx) illumination. Average processing time, θ, and FPS were calculated for each frame selected. The error angles in different illumination are shown in Figures 15, 16, respectively. The performance of this algorithm is shown in Table 1.

**Table 1:** The performance of different methods in indicators of time,error angle, accuracy, and FPS compared with those in the literature.

**Methods**	**Average time(s)**	**Maximum angle(θ)**	**Minimum angle(θ)**	**Average angle(θ)**	**Accuracy (%)**	**FPS**
This study	312.3	3.88	1.04	1.49	98.6	4.4
Algorithm-1	1000.2	12.82	8.69	8.36	70.1	2.1
Algorithm-2	828.4	9.24	5.88	6.89	88.5	3.8
Algorithm-3	459.9	4.95	3.28	3.78	93.6	2.9
Algorithm-4	610.4	10.4	7.77	8.59	84.9	2.6

To further verify the reliability and real-time performance of this study it is compared with the methods proposed by Hough (1962) (Hough Transform) (Algorithm-1), Ji and Qi (2011) (Algorithm-2), Zhou et al. (2021) (Algorithm-3), and Zhang et al. (2018b) (Algorithm-4). Additionally, we will analyze the results of the performance among different methods in terms of accuracy and processing time. The Hough transform is a very classical algorithm. After the images are segmented using the pre-processing method proposed, we used the Hough transform to detect the crop rows and record the data. Algorithm-2 mapped the coordinates of the image space to the parameter space through random numbers. The parameter space feature points disappeared when dealing with the case of divergence of tassels, resulting in significant deviations in the Hough line detection process, and the navigation line's average error angle reached 6.89°. Moreover, Algorithm-2 used the Hough transform algorithm, which had a higher computational cost, and the average time to process a frame was more than 800 ms, which could not meet the real-time requirements of field navigation. In response to the problems of Algorithm-2, the proposed algorithm took the left and suitable regions of the image and obtained the multi-ROI by sliding off the bounding box to extract the feature points, which effectively calculated the navigation line under the dense conditions of crop rows distribution. The average error angle of the navigation line calculated by Algorithm-3 is 3.78°. This error is because the ROI window proposed in the paper could not completely cover the tassels after the ROI window was slid upward when the tassels were bifurcated during the extraction process, which led to deviations in the fitting. Furthermore, the algorithm selects the appropriate feature points by the center of each ROI. However, the navigation lines were biased due to the problems of forked tassels. The algorithm in this article deals with the deviation points by fixed-size ROI and the ROI optimization to extract the tassels completely. Algorithm-4 removed feature points by selecting the midpoints of the left and right edge points in the image strips. This approach had better performance when dealing with small target plants, but when dealing with more oversized tassels, especially the characteristic tassels with bifurcation, the feature points calculated in this way did not express the crop distribution in this region, resulting in a significant error in the subsequent detection lines extraction. The proposed algorithm extracted feature points more completely by dividing micro-ROIs, avoiding the above problems. By comparison, the algorithm of this study has high accuracy and real-time performance in crop row detection in maize fields at the tasseling stage.

**Figure 17:**
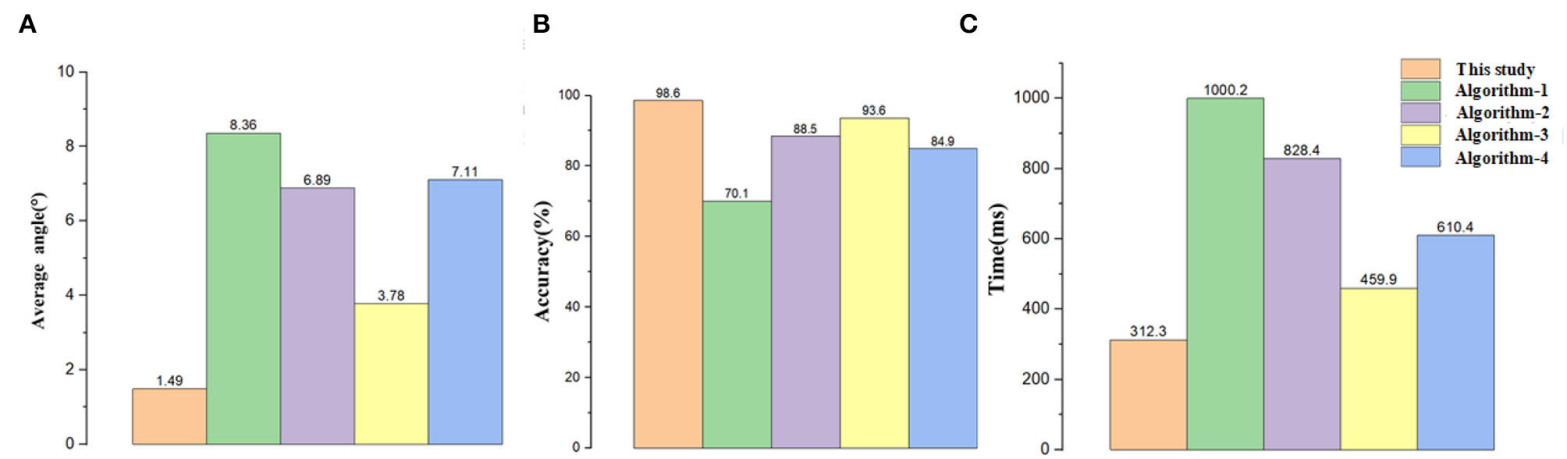
Results of comparative data. **(A)** The data of angle, **(B)** the data of accuracy, and **(C)** the data of time.

**Figure 18:**
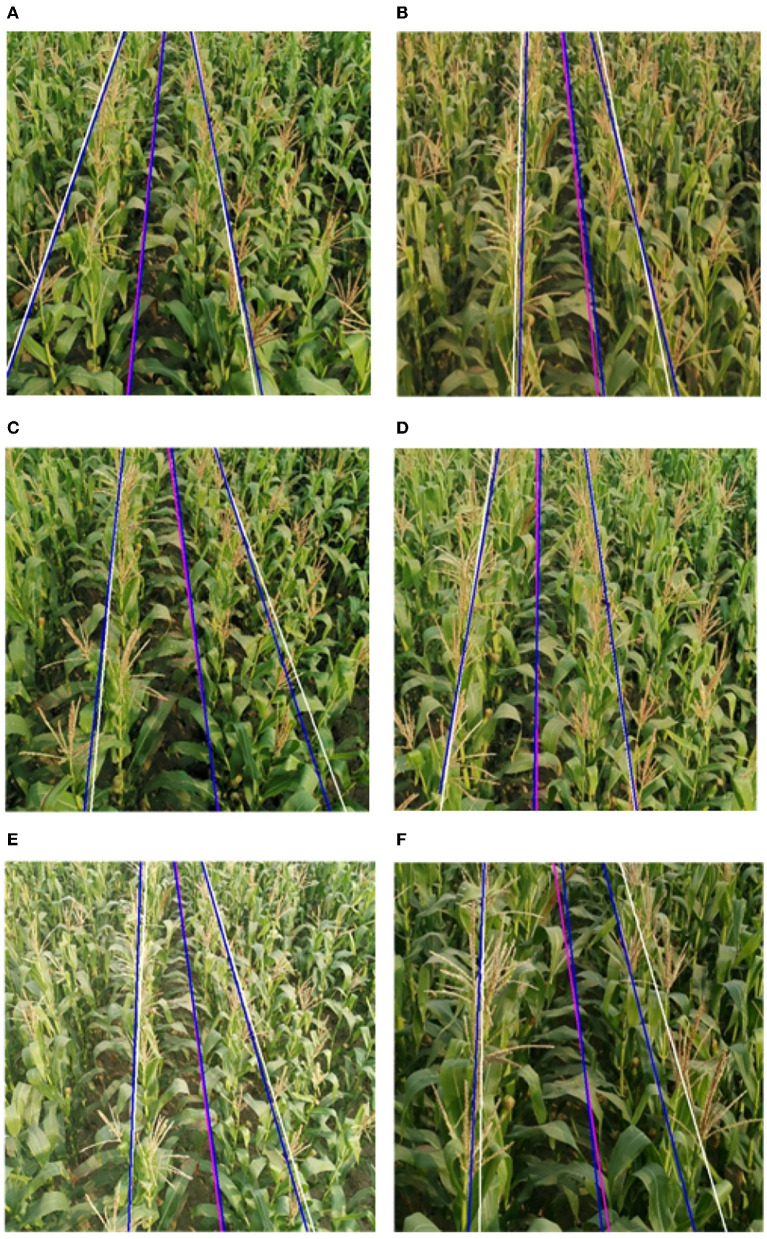
Experimental results of crop rows detection. The blue lines are the manual calibration lines, the white lines are the algorithmic crop rows identification lines, and the pink lines are the algorithmic navigation lines. **(A)** Type-1 field, **(B)** Type-2 field, **(C)** Type-3 field, **(D)** Type-4 field, **(E)** Type-5 field, and **(F)** Type-6 field.

**Figure 19:**
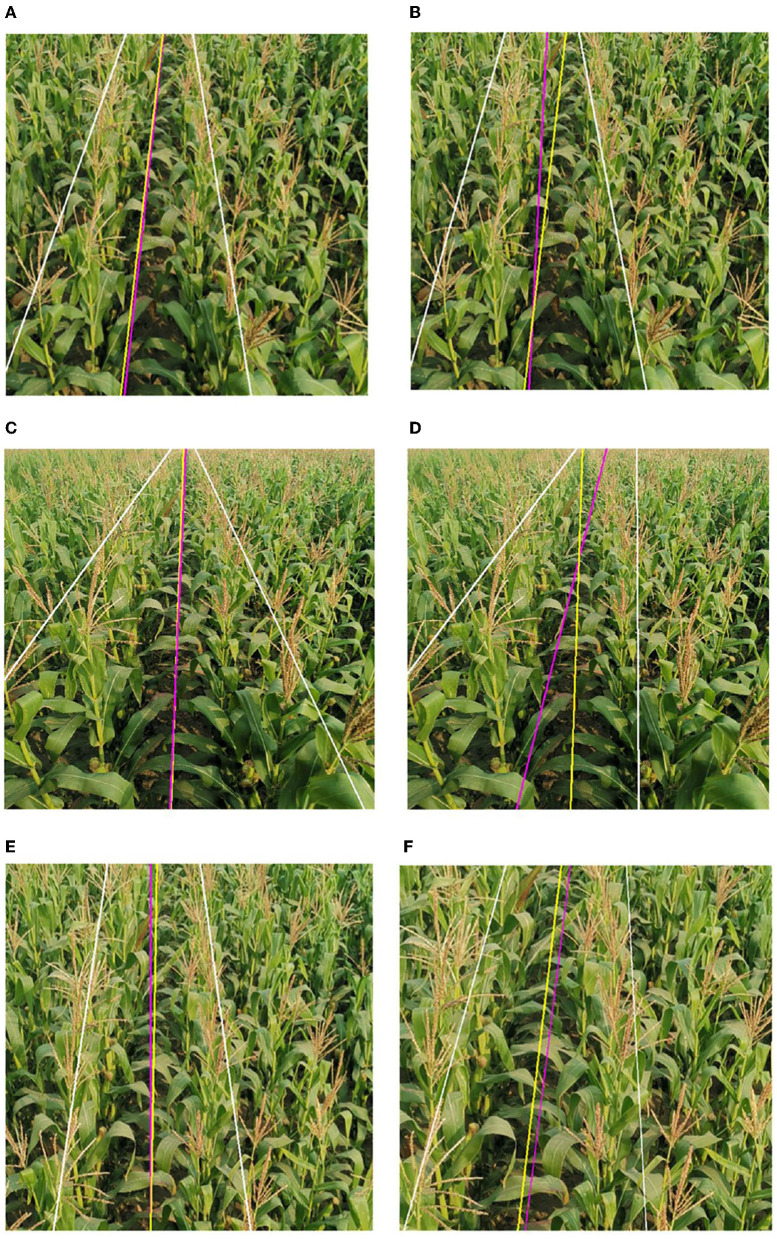
Extraction results comparison between literature and algorithm in this article. This algorithm's crop rows detection lines are white, the manually calibrated navigation line is yellow, and the corresponding algorithm's navigation line is pink. **(A)** Algorithm of this paper, **(B)** Algorithm-2, **(C)** Algorithm of this paper, **(D)** Algorithm-3, **(E)** Algorithm of this paper, and **(F)** Algorithm-4.

The performance of each algorithm is shown in Table 1. The main parameters are shown in Figure 17 so that they can be more intuitive. Compared to current popular algorithms, we have improved accuracy by at least 5% and single-frame processing time by at the least 62.3%. The processing results of this algorithm are shown in Figure 18. The results of the above comparison experiment are shown in Figure 19.

## 4. Conclusion and Future Study

Based on machine vision, we propose a real-time method for the extraction of navigation lines in the maize field during the tasseling stage. Field navigation line extraction in the maize crop rows during the tasseling phase is challenging because the tassels are hard to be segmented from the background, and extracting their information is difficult. We propose a real-time crop rows detection algorithm based on logarithmic transformation and micro-ROI in the field during the tassel period to address these issues. After cutting the image captured by the CMOS camera (RGB) to obtain a 600 × 600 (pix) image, the algorithm performs a logarithmic transformation to augment the distinction between the tassel and the background. This research converted the image to the HSV color space. Additionally, the background of the tassels was created as a mask. After the lower part of the image is divided into eight horizontal strips, we use the bounding box to determine the initial ROI by selecting the starting point. The final result of the initial ROI is determined by updating its bounding box position. The current bounding box is slid along the negative direction of the y-axis of the image coordinate system in steps Δ*h* and updated until the multi-ROI is reached. The ROI is divided into cells to get the micro-ROIs, and then the feature points are extracted. Feature points are fitted to derive crop row detection lines, on which the navigation lines are then calculated. We fit the feature points to make crop rows detection and navigation line. The error in the navigation line of this algorithm is stable at 1.49°, and the average computational time of the single frame is 312.3 ms. The accuracy is reaching 98.6%. After the Comparison experiment, the algorithm proves to have a clear advantage in terms of real-time and accuracy.

However, there are still some limitations to this method, such as different climates and different crop row spacing, which can reduce the accuracy of the algorithm. In the future, we will focus on new methods of feature extraction (Xue et al., 2021b), image augments (Sui et al., 2020), and ROI adaptability to segment a variety of tasseled plants and calculate adaptive ROI with a wide range of planting rows. In addition, how to apply the energy-efficient spiking neural networks to crop rows detection is another interesting topic. Because the SNNs hold the potential to provide a good performance equivalent to that of DNNs while with low latency and high computational efficiency (Feldmann et al., 2019; Zhang et al., 2021; Luo et al., 2022).

## Data Availability Statement

The original contributions presented in the study are included in the article/supplementary material, further inquiries can be directed to the corresponding author.

## Author Contributions

YZ and YYa performed all the experiments. All authors contributed to the design of the experiment, result interpretation, and writing.

## Funding

The authors acknowledge that the research was financially supported by the Nation Key Research and Development Program of China (Grant No. 51905004), the University Synergy Innovation Program of Anhui Province (Grant No. GXXT-2020-011), Central Leading Local Science and Technology Development Special Foundation for Sichuan Province PR China (No. 2021ZYD0020), and Scientific Research Project of Yibin University (No. 0219024502).

## Conflict of Interest

DL was employed by JD AI Research. The remaining authors declare that the research was conducted in the absence of any commercial or financial relationships that could be construed as a potential conflict of interest.

## Publisher's Note

All claims expressed in this article are solely those of the authors and do not necessarily represent those of their affiliated organizations, or those of the publisher, the editors and the reviewers. Any product that may be evaluated in this article, or claim that may be made by its manufacturer, is not guaranteed or endorsed by the publisher.
